# Media Coverage, Forecasted Posttraumatic Stress Symptoms, and Psychological Responses Before and After an Approaching Hurricane

**DOI:** 10.1001/jamanetworkopen.2018.6228

**Published:** 2019-01-04

**Authors:** Rebecca R. Thompson, E. Alison Holman, Roxane Cohen Silver

**Affiliations:** 1 Department of Psychological Science, University of California, Irvine; 2 Sue & Bill Gross School of Nursing, University of California, Irvine; 3 Program in Public Health, University of California, Irvine; 4 Department of Medicine, University of California, Irvine

## Abstract

**Importance:**

Exposure to disaster-related media coverage is associated with negative mental health outcomes. However, risk factors that render individuals vulnerable to this exposure are unknown. Hurricane-associated media exposure was expected to explain the association between forecasted posttraumatic stress (PTS) and adjustment after the hurricane.

**Objective:**

To examine forecasted PTS responses and media coverage as risk factors for negative mental health outcomes in the context of media coverage of an approaching disaster (Hurricane Irma).

**Design, Setting, and Participants:**

In a representative probability community sample of 1637 adults from Florida, respondents completed 2 online surveys: the first during the 60 hours before Hurricane Irma’s landfall (wave 1; September 8-11, 2017) and the second approximately 1 month later (wave 2; October 12-29, 2017). Poststratification weights were applied to facilitate population-based inferences. Data were analyzed from October 19 through 31, 2018.

**Main Outcomes and Measures:**

Posttraumatic stress responses, psychological distress, functional impairment, and worry about future events.

**Results:**

The wave 1 survey included 1637 participants (57.0% response rate); 1478 participants were retained at the wave 2 follow-up (90.3% retention) (weighted proportion of women, 62.2%; mean [SD] age, 59.1 [15.2] years). The final weighted sample closely approximated US Census benchmarks for the state of Florida. Data analyses using structural equation modeling revealed that exposure to media coverage of the hurricane (β = 0.21; 95% CI, 0.11-0.31; *P* < .001) and forecasted PTS (β = 0.44; 95% CI, 0.35-0.52; *P* < .001) were significantly associated with adjustment after the hurricane. In addition, a significant indirect path from forecasted PTS to adjustment after the storm occurred through exposure to hurricane-related media coverage (β = 0.07; 95% CI, 0.05-0.08; *P* < .001). Covariates included demographics, mental health diagnoses before the storm, perceived evacuation zone status, and degree of hurricane exposure.

**Conclusions and Relevance:**

Results of this study provide a more thorough understanding of how psychological factors before hurricanes are associated with adjustment after hurricanes via media consumption. The findings may also demonstrate the importance of considering prestorm psychological factors when assessing poststorm outcomes, with implications for the media and public health efforts.

## Key Points

**Question:**

Do forecasted posttraumatic stress symptoms play a role in the association between media exposure to an approaching hurricane and psychological outcomes after the storm?

**Findings:**

In this longitudinal online survey of a representative sample of 1478 Florida residents, disaster-related media exposure partially accounted for the association between forecasted posttraumatic stress and psychological outcomes in the aftermath of Hurricane Irma.

**Meaning:**

Forecasted posttraumatic stress symptoms experienced before a community trauma presage media consumption and subsequent mental health outcomes, with important implications for the media and the public’s mental health.

## Teaser

This online survey study examines the association of forecasted posttraumatic stress symptoms and negative mental health outcomes in the context of exposure to media coverage of Hurricane Irma as an approaching disaster among a weighted sample of Florida residents.

## Introduction

For coastal communities, hurricanes are increasingly common weather hazards that can cause major destruction and death. Days before a major hurricane, life is disrupted as individuals prepare their homes and evacuate if necessary. The psychological effect of these events may also be severe; exposure is often associated with posttraumatic stress (PTS) symptoms and other mental health conditions.^1,2^ One study of affected populations after Hurricane Katrina found PTS prevalence as high as 30% and almost 50% prevalence for anxiety disorders.^3^ However, urgent conduct of research with disaster-threatened populations is difficult,^4^ so we know little about how the psychological experience of individuals anticipating a disaster may influence their subsequent responses. Given that the science surrounding global climate change anticipates increasing hurricane activity, understanding how populations at risk for hurricane exposure respond to the threat of disaster is important.

The news media is an important information source for many in the path of these storms. In the past, individuals relied heavily on local television news reports for storm-related information,^5^ but online media sources now often supplement disaster reports from official sources.^6^ Although information-seeking behavior may be a rational response among community members facing an evolving hazard, decades of research on media exposure to trauma suggests that extensive media consumption during a disaster event is often associated with negative consequences. Indeed, there is evidence of a link between disaster-related media consumption and negative psychological outcomes, including PTS.^7^ Specifically, use of television^8^ and social media^9^ in the aftermath of hurricanes has been linked to increased PTS and depression. However, natural disasters account for a much smaller proportion of the literature on this topic relative to studies conducted after man-made or technological disasters.^7^ Also, to date, no research on consumption of media during and shortly after an impending disaster, such as a hurricane, has been conducted.

Individuals’ anticipated response to a disaster is an important factor that may influence their media consumption surrounding the threat and their subsequent responses. People often predict how they might feel in the future through a process termed *affective forecasting*.^10^ A tendency toward negative emotional forecasts, or negative future orientation, has been associated with increased PTS^11^ and psychological distress^12^ in the aftermath of a community trauma. People also tend to make more negative attributions about future negative events than they do about past negative events.^13^ Furthermore, pretraumatic stress, or intrusive thoughts or images about negative future events, was strongly associated with subsequent PTS symptoms in a sample of Danish soldiers from before to after deployment.^14^ Together, these findings suggest that individuals’ forecasted PTS responses in the days leading up to a hurricane may be associated with more negative mental health outcomes in its aftermath.

In addition, some individuals are more likely than are others to forecast greater PTS responses in anticipation of an impending disaster. Most people are not particularly accurate when predicting their future emotional responses, especially when it comes to predicting the durability of their responses to negative events.^15^ However, individuals with higher levels of depression and anxiety reliably forecast more negative emotional responses to future events.^16,17^ At the same time, forecasted PTS is also likely to be associated with increased exposure to hurricane-related media coverage. Prior research suggests that anxious^18^ and healthy^19,20^ individuals tend to orient toward stimuli that they find threatening. Similarly, through a process known as *uncertainty management*, individuals who are worried about a particular event may try to assuage their anxiety by seeking out information related to that event.^21^ However, if individuals choose to mitigate their hurricane-related anxiety by seeking storm-related information in the media, increased anxiety may result instead.^7^ Therefore, these individuals may be vulnerable to a cycle of increased media consumption and psychological distress in the aftermath of a disaster. Thus, forecasted PTS may also be indirectly associated with outcomes through disaster-related media consumption.

### The Present Study

The 2017 Atlantic hurricane season was the most active in more than a decade, producing 17 named storms, 10 of which became hurricanes.^22^ These hurricanes included the first major hurricanes to hit the mainland United States in more than a decade, including Hurricane Irma, one of the strongest Atlantic Ocean hurricanes ever recorded. Hurricane Irma was a category 5 storm at its strongest but weakened to a category 3 storm before making landfall on the US mainland around 3:30 pm on September 10, 2017. The storm killed 92 persons in the United States (with 42 additional deaths in Caribbean nations) and caused approximately $50 billion of damage.^23^ Media provided 24-hour sensationalized coverage, which described the possibility of “a catastrophic hit” and “worse than feared” destruction.^24^ News reports featured reporters standing in high winds and rain to illustrate the dire conditions outside.^25^ The media broadcasted this coverage nationally, not just locally, thus expanding the disaster’s reach beyond directly affected communities.

This storm also had an uncertain path, which shifted across Florida in the days preceding landfall. Indeed, at 1 point, the entire state was threatened. This possibility presented a unique research opportunity: we studied the association between anticipated responses to an impending disaster and actual responses in its aftermath by collecting data from a representative sample of Florida residents immediately before and soon after the hurricane made landfall. This process allowed us to examine how responses to the storm evolved from before to after the hurricane across the state. We hypothesized that forecasted PTS responses to Hurricane Irma would be associated with increased hurricane-related media consumption, which in turn would be associated with poorer poststorm adjustment, after controlling for demographics, prior mental health status, and objective indicators of storm exposure. In particular, we were interested in poststorm PTS, which captures event-specific stress responses; psychological distress, which captures generalized stress responses; functional impairment, which captures mental and physical health effects on daily functioning; and worry about the future, which captures future-oriented concerns. Each of these indicators assessed a unique component of postdisaster adjustment.

## Methods

### Participants, Design, and Procedures

Participants came from the GfK KnowledgePanel (GfK Custom Research North America), a national panel of adult US residents recruited via address-based sampling to answer Web-based surveys in exchange for Internet access and other compensation. All panelists from Florida were recruited to participate in a study about their responses to the impending Hurricane Irma, which was approaching Florida as a category 4 storm after making landfall in Cuba with category 5 wind speeds. This study followed the American Association for Public Opinion Research (AAPOR) reporting guideline. The institutional review board of the University of California, Irvine approved all procedures, with implicit consent from all respondents.

Beginning at 6 pm on September 8, 2017, GfK sent 2873 panelists a link to an online survey they could complete on a computer, tablet, or smartphone; 1637 completed it (57.0% participation rate). The survey included individuals’ perceived evacuation status and forecasted psychological responses to the storm. Surveys were available for completion until 3 pm on September 11, 2017; 1555 respondents (95.0%) completed the wave 1 survey within the first 48 hours.

Approximately 1 month later (October 12 through 29, 2017), GfK fielded a second survey to all wave 1 participants and those panelists from Florida who had previously participated in a national study of US residents’ responses to the Boston Marathon bombing^26^ (n = 1723). Of these, 1518 participants (87.9%) completed the wave 2 survey, which included questions about participants’ psychological and social functioning since the storm, their media consumption about it, and the degree to which they were affected by the storm’s landfall. The final sample of individuals who completed both surveys consisted of 1478 individuals (90.3% retention from wave 1). GfK provided poststratification weights for all participants to account for discrepancies between the sample and US Census benchmarks for Florida. At both waves, respondents provided consent by completing the surveys after reading a brief introduction describing the study.

### Measures

#### Demographics and Mental Health Diagnoses

Before wave 1, all panelists reported demographic characteristics (ie, age, sex, educational attainment, and ethnicity) and mental health history. Participants indicated whether a physician had ever given them a diagnosis of an anxiety or a depressive disorder; responses were coded as 0 (neither), 1 (either anxiety or depression), or 2 (both anxiety and depression).

#### Perceived Evacuation Zone Status

At wave 1, perceived evacuation zone status was calculated based on participants’ responses to 2 questions. Participants who reported evacuating and those who believed they were in an evacuation zone were coded as 1; participants who reported not evacuating because they did not believe they were in an evacuation zone were coded as 0.

#### Hurricane Irma Direct Exposure

At wave 2, participants reported on a 9-item scale the degree to which they were directly exposed to Hurricane Irma. Participants could report staying in their home while under evacuation order, experiencing damage to their home or property, personal injury, or knowing someone who was injured or killed in the storm. Responses to this scale were dichotomized for analyses.

#### Hurricane Irma Media Exposure

At wave 2, participants reported the mean number of hours per day they spent engaging with 3 media sources “in the days during and following the recent hurricanes,” including traditional media (ie, television, radio, and print news), online news sources (CNN, Yahoo, NYTimes.com, etc), and social media (Facebook, Twitter, Reddit, etc). Participants could report up to a maximum of 11 hours per day for each source; owing to the possibility of simultaneous exposure across multiple media platforms, respondents could report a maximum of 33 hours per day across all sources.

#### PTS Symptoms

At both waves, PTS symptoms were measured using the Primary Care PTSD (Posttraumatic Stress Disorder) Screen for *Diagnostic and Statistical Manual of Mental Disorders* (Fifth Edition) (*DSM-5*).^27^ The 5-item scale assessed the severity of symptoms corresponding to the *DSM-5* posttraumatic stress disorder symptom clusters on a modified Likert-type scale ranging from 1.00 to 5.00 (with higher scores indicating more frequent symptoms). At wave 1, participants were asked: “With respect to Hurricane Irma and its aftermath, how often do you think you *will* experience [these symptoms] a *week or two from now*?” At wave 2, they were asked to report how often they had experienced these symptoms with respect to Hurricane Irma during the previous week. At both points, this scale maintained good internal reliability (wave 1 α = 0.86; wave 2 α = 0.87).

#### Psychological Distress

General psychological distress (ie, anxiety, depression, somatization, and anger) was measured at wave 2 using 13 items identified through factor analysis in previous studies, including 9 items drawn from the Brief Symptom Inventory 18^28^ and 4 anger items from the original 53-item Brief Symptom Inventory,^29^ each assessed on a Likert-type scale ranging from 1.00 to 4.00 (greater scores indicate greater distress). The measure maintained excellent internal reliability in this sample (α = 0.92); the mean was calculated to create an index of psychological distress.

#### Functional Impairment

Functional impairment was assessed at wave 2 using 4 items modified from the 36-Item Short Form Health Survey.^30^ These items assessed the extent to which participants’ mental and physical health interfered with work and social functioning on a Likert-type scale ranging from 1.00 to 5.00 (higher scores indicated greater impairment), which maintained good internal reliability in this sample (α = 0.86).

#### Worry About Future Events

Worry was assessed at wave 2 using 8 items adapted from those used in prior research after September 11, 2001,^31,32^ that assessed worries in the previous week about the likelihood of being exposed to natural disasters, environmental hazards, violence, and economic hardship in the future. Scores ranged from 1.00 to 5.00, with higher scores indicating greater worry about future events. These items maintained excellent internal consistency in this sample (α = 0.90). The mean was calculated to create a composite worry score for each participant.

### Statistical Analysis

Analyses were conducted using Stata software (version 14.2; StataCorp) from October 19 through 31, 2018. For all tests, significance was measured at *P* < .05, and all *P* values were 2-sided. To assess the association with forecasted PTS responses and media exposure on outcomes, a series of structural equation models were constructed using Stata’s structural equation model builder. This analysis incorporates several regression analyses simultaneously, enabling testing of possible causal pathways over time. First, a measurement model was constructed for the latent variable wave 2 adjustment, which consisted of the variables for posthurricane PTS, psychological distress, functional impairment, and worry about future events. Next, we developed a theoretical model to test the associations among media exposure to Hurricane Irma, forecasted PTS responses, and posthurricane adjustment, controlling for covariates. The initial model included the basic mediation model, with covariates included at the most exogenous level. Covariates for wave 1 forecasted PTS included age, sex, educational attainment (Bachelor’s degree or greater vs other), ethnicity (white non-Hispanic vs other), perceived evacuation zone status, and prior mental health diagnoses. Further ethnic breakdowns were tested but did not reveal any significant differences on outcomes. Based on theoretical considerations, additional paths were drawn from prior mental health diagnoses to wave 2 PTS and from perceived evacuation zone status to hurricane-related media exposure; the latter path was not significant and was not included in the final model. Finally, an additional path from wave 2 direct hurricane exposure to wave 2 adjustment was added. Stata’s structural equation model builder also allows for the inclusion of sampling weights, which were used in all models to facilitate population inferences. Analyses were conducted with and without the poststratification weights; the pattern of results remained the same. To retain sample representativeness, all statistics, including percentages reported herein, were analyzed using poststratification weights.

Goodness of fit was assessed using the coefficient of determination (CD) and the standardized root mean square residual (SRMR), which are most appropriate for weighted survey data.^33^ The CD is a representation of the percentage of variance in the dependent variable that the model explains and may be interpreted similarly to an *R*^2^ value in linear regression. For the SRMR, a value of less than 0.08 indicates good model fit.

## Results

Table 1 presents the final weighted and unweighted descriptive statistics for study-related variables. The final weighted sample of 1478 participants (558 men [37.8%; weighted proportion, 44.6%] and 920 women [62.2%; weighted proportion, 55.4%]; mean [SD] age, 59.1 [15.2] years) closely approximated US Census benchmarks for Florida. Media exposure in the sample was high, with participants reporting a mean of 7.45 (SD, 6.93; weighted mean, 8.12 [standard error {SE}, 0.31]) hours of media exposure per day across sources. Specifically, participants reported a mean (SD) of 4.04 (3.44) (weighted mean, 4.07; SE, 0.14) daily hours of television, radio, and print news; 1.95 (2.76) (weighted mean, 2.19; SE, 0.12) daily hours of online news; and 1.53 (2.63) (weighted mean, 1.93; SE, 0.12) daily hours of social media in the days during and after Hurricane Irma.

**Table 1.** Descriptive Statistics for Variables of Interest^a^

Variable	Unweighted No. (%)	Weighted No. (%)^b^
Sex, No. (%)		
Male	558 (37.8)	652.8 (44.6)
Female	920 (62.2)	809.6 (55.4)
Ethnicity/race, No. (%)		
Non-Hispanic white	1163 (78.7)	907.5 (62.1)
Black or African American	103 (7.0)	168.1 (11.5)
Non-Hispanic other	56 (3.8)	70.2 (4.8)
Hispanic	156 (10.6)	316.5 (21.6)
Educational attainment, No. (%)		
<High school	23 (1.6)	71.6 (4.9)
High school diploma or equivalent	230 (15.6)	497.9 (33.4)
Some college or Associate’s degree	534 (36.1)	474.1 (32.4)
≥Bachelor’s degree	691 (46.8)	428.8 (29.3)
Household income, No. (%)		
<$25 000	230 (15.6)	254.4 (17.4)
$25 000-$49 999	371 (25.1)	369.4 (25.3)
$50 000-$74 999	311 (21.0)	302.2 (20.7)
$75 000-$99 999	263 (17.8)	227.6 (15.6)
≥$100 000	303 (20.5)	308.8 (21.1)
Mental health (anxiety or depression) diagnoses, No. (%)		
0 (neither)	1223 (82.8)	1218.0 (83.3)
1 (either)	176 (11.9)	166.2 (11.4)
2 (both)	79 (5.4)	77.9 (5.3)
Perceived evacuation zone status, No. (%)		
Yes	791 (53.6)	783.1 (53.7)
No	684 (46.4)	675.0 (46.3)
Direct hurricane exposure, No. (%)		
Yes	998 (67.5)	986.4 (67.5)
No	480 (32.5)	476.0 (32.6)
**Variable**	**Unweighted Mean (SD) [Range]**	**Weighted Mean (SE)**^b^
Age, y	59.1 (15.2) [18-91]	51.7 (0.7)
Anticipated PTS response (wave 1)^c^	1.81 (0.8) [1.00-5.00^d^]	1.84 (0.03)
Hurricane-related media exposure (wave 2), h/d^e^	7.45 (6.93) [0-33.00]	8.12 (0.31)
PTS response	1.46 (0.67) [1.00-5.00^d^]	1.49 (0.03)
Psychological distress	0.44 (0.58) [0-4.00^f^]	0.50 (0.03)
Functional impairment	1.55 (0.82) [1.00-5.00^g^]	1.58 (0.03)
Worry about future events	2.03 (0.80) [1.00-5.00^h^]	2.10 (0.04)

Abbreviations: PTS, posttraumatic stress; SE, standard error.

^a^ Includes 1478 respondents. Percentages have been rounded and may not total 100.

^b^ Weights adjust estimates for sampling design and poststratification to US Census benchmarks.

^c^ Measured from September 8 through 11, 2017.

^d^ Higher scores indicate more symptoms.

^e^ Measured from October 12 through 29, 2017.

^f^ Higher scores indicate greater distress.

^g^ Higher scores indicate greater functional impairment.

^h^ Higher scores indicate greater worry about future events.

Table 2 presents the correlations among the variables included in the model. The 4 dependent variables of interest were correlated with one another (variables 10-13 in the correlation matrix; correlations ranged from *r* = 0.53 to *r* = 0.72), which was expected given that they all represent a type of negative psychological outcome. For this reason, despite the conceptual distinctness of these constructs, the 4 variables were combined into 1 latent construct of posthurricane adjustment in subsequent structural equation model analyses. All variables were also tested individually in separate path models; the patterns of responses remained identical and significant for each outcome.

**Table 2.** Correlations Among the Variables Included in the Model

Variable by Number^a^	1	2	3	4	5	6	7	8	9	10	11	12	13
1. Age	1.00												
2. Female	−0.09^b^	1.00											
3. College degree	−0.10^b^	−0.09^b^	1.00										
4. White ethnicity	0.33^b^	−0.04	−0.01	1.00									
5. Mental health diagnosis	−0.07^c^	0.11^b^	−0.05^d^	0.00	1.00								
6. Perceived evacuation zone (yes or no)	0.03	−0.03	−0.03	0.03	0.07^c^	1.00							
7. Forecasted PTS	−0.12^b^	0.13^b^	−0.01	−0.08^b^	0.17^b^	0.15^b^	1.00						
8. Media exposure	−0.11^b^	0.08^c^	−0.11^b^	−0.13^b^	0.10^b^	0.07^d^	0.32^b^	1.00					
9. Direct exposure	−0.10^b^	0.00	−0.02	−0.04	0.06^d^	0.37^b^	0.16^b^	0.11^b^	1.00				
10. Wave 2 PTS	−0.08^c^	0.10^b^	−0.06^d^	−0.12^b^	0.24^b^	0.15^b^	0.50^b^	0.32^b^	0.25^b^	1.00			
11. Wave 2 psychological distress	−0.18^b^	0.10^b^	−0.06^d^	−0.14^b^	0.33^b^	0.13^b^	0.37^b^	0.26^b^	0.17^b^	0.68^b^	1.00		
12. Wave 2 functional impairment	−0.04	0.11^b^	−0.08^c^	−0.08^c^	0.34^b^	0.13^b^	0.29^b^	0.23^b^	0.16^b^	0.58^b^	0.72^b^	1.00	
13. Wave 2 worry	−0.15^b^	0.14^b^	−0.05^d^	−0.13^b^	0.23^b^	0.14^b^	0.47^b^	0.33^b^	0.22^b^	0.71^b^	0.63^b^	0.53^b^	1.00

Abbreviation: PTS, posttraumatic stress.

^a^ Variables 1 to 5 were collected before wave 1; variables 6 and 7, during wave 1 (before the storm, September 8-11, 2017); and variables 8 to 13, during wave 2 (after the storm, October 12-29, 2017). Descriptions of these variables and how they were measured are given in the Measures subsection of the Methods section.

^b^ *P* < .001.

^c^ *P* < .01.

^d^ *P* < .05.

**Figure 1.**
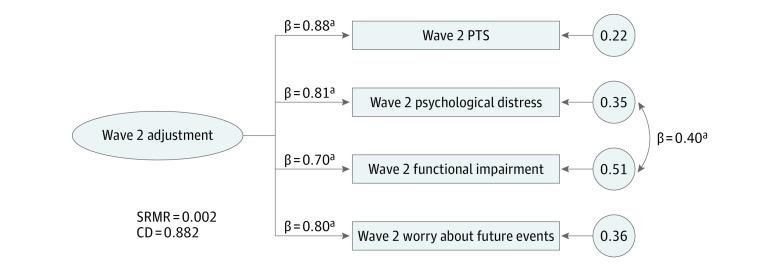
Final Measurement Model for Wave 2 Adjustment Posttraumatic stress (PTS), psychological distress, functional impairment, and worry about future events were all measured at wave 2 (October 12 through 29, 2017) approximately 1 month after Hurricane Irma; analyses include 1446 respondents. Descriptions of these variables and how they were measured are given in the Measures subsection of the Methods section. Values above the arrows are standardized regression coefficients; values inside the circles are standardized error terms. CD indicates coefficient of determination; SRMR, standardized root mean square residual. ^a^*P* < .001.

**Figure 2.**
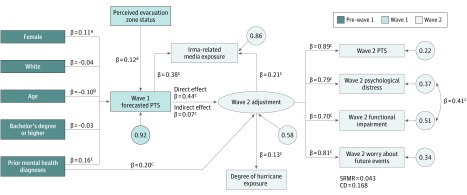
Structural Equation Model Predicting Wave 2 Adjustment Wave 1 forecasted posttraumatic stress (PTS) was assessed from September 8 through 11, 2017; wave 2 outcomes, from October 12 through 29, 2017. Analyses include 1446 respondents. Descriptions of these variables and how they were measured are given in the Measures subsection of the Methods section. Values above the arrows are standardized regression coefficients; values inside the circles are standardized error terms. CD indicates coefficient of determination; SRMR, standardized root mean square residual. ^a^*P* < .01. ^b^*P* < .05. ^c^*P* < .001.

Figure 1 presents the final measurement model for the latent construct of wave 2 adjustment. In the initial model, which included only the 4 observed outcome variables loading onto 1 latent variable, all factor loadings were high (≥0.77), and model fit was good (SRMR = 0.031; CD = 0.895). Model fit was improved by adding an additional covariance path between the error terms for psychological distress and functional impairment, the 2 most correlated outcomes (SRMR = 0.002; CD = 0.882). This measurement model was then expanded to create the final theoretical model, which is presented in Figure 2.

In the final model, wave 1–forecasted PTS responses were significantly associated with hurricane-related media exposure (β = 0.38; 95% CI, 0.29-0.46; *P* < .001) and wave 2 adjustment (β = 0.44; 95% CI, 0.35-0.52; *P* < .001), controlling for all covariates. Perceived evacuation zone status was significantly associated with wave 1–forecasted PTS responses (β = 0.12; 95% CI, 0.05-0.20; *P* = .002) but not hurricane-related media exposure. Prior mental health diagnoses were associated with wave 1–forecasted PTS responses (β = 0.16; 95% CI, 0.08-0.24; *P* < .001) and wave 2 adjustment (β = 0.20; 95% CI, 0.12-0.29; *P* < .001). In alternative models, a path between prior mental health diagnoses and hurricane-related media exposure was included; this path was not significant, and so the more parsimonious model was chosen. Hurricane-related media exposure was significantly associated with wave 2 adjustment (β = 0.21; 95% CI, 0.11-0.31; *P* < .001), controlling for direct exposure to the hurricane (β = 0.13; 95% CI, 0.05-0.21; *P* < .001). In addition, the indirect path from wave 1–forecasted PTS responses to wave 2 adjustment through hurricane-related media exposure was significant, although relatively small in magnitude (β = 0.07; 95% CI, 0.05-0.08; *P* < .001). This model was a strong fit for the data (SRMR = 0.043; CD = 0.168) (Figure 2). All associations were significant in the expected directions.

## Discussion

Our findings indicate that forecasted PTS responses and storm-related media consumption before an approaching hurricane are important correlates of poststorm psychological adjustment. Forecasted PTS is also indirectly linked to poststorm outcomes via consumption of disaster-related media coverage, even when controlling for direct storm exposure. In fact, forecasted PTS responses were associated with increased media consumption, but perceived evacuation zone status was not, meaning that prestorm psychological factors appear to play a more important role in media engagement surrounding a disaster than is typically acknowledged. Given that this media engagement during a disaster has been associated with negative psychological consequences and downstream implications for physical health,^34^ understanding the predictors associated with this behavior is of particular importance. Furthermore, it appears that individuals’ prestorm vulnerability to distress, as measured by forecasted PTS, increases media exposure, with significant implications for postdisaster adjustment.

These results are also important because they represent the first attempt by researchers to analyze how prestorm psychological factors are associated with subsequent adjustment through prospective analyses with surveys fielded in the days leading up to a hurricane. Because we are not relying on retrospective reports of participants’ psychological functioning or storm perceptions, which can be influenced by situational factors^35^ or degrade over time,^36^ we can be more confident in the ecological validity of our findings. Furthermore, the study design, which involved sampling from within a statewide panel and the use of sampling weights to adjust for probability of inclusion into the study, enables us to extrapolate from these findings to make population inferences.

### Future Directions

Several questions remain unanswered. For example, because the use of online social media for updates during a developing crisis is associated with greater distress responses relative to other media sources,^37^ perhaps greater social media use during an approaching hurricane has a stronger association with poststorm outcomes when compared with traditional media. Poststorm responses to media may also be differentially associated with prestorm psychological projections. Preliminary analyses of our data suggest this is not the case; however, this association should be examined using stronger measures of media exposure, ideally in real time.

### Limitations

Despite use of a statewide panel and population weights, we acknowledge that the present sample is not necessarily representative of Florida residents. GfK sent invitations to participate in the wave 1 survey to all their Florida panelists to capture as much data on Floridians’ responses as possible. However, this sampling design precluded our ability to oversample in harder-to-recruit populations. Furthermore, the panel is designed to recruit samples that are demographically representative of the populations from which they are drawn, but this does not include geographic representation within smaller communities. As a result, the geographic distribution of participants in our sample does not necessarily mirror that of Florida. This is important for studies of natural hazards because the geographic distribution of the sample may not be representative in terms of population hurricane exposure, objectively via strong winds and storm surge as well as subjectively via local media reports. Sampling weights can correct for discrepancies between the sample and census benchmarks, but it would be helpful for future research to improve geographic representation as well.

We also acknowledge the possibility of a Hawthorne effect in our sample, such that asking individuals at wave 1 to attend to their expectations for future distress may have amplified reports of distress at wave 2. This effect can be a concern in longitudinal survey research because participants’ continued participation in surveys introduces the possibility that their previous responses may affect subsequent behavior. However, in this case, we see no indication of the Hawthorne effect occurring. When comparing participants who did not participate in wave 1 with those who did using bootstrapped *t* tests, there are no differences in wave 2 adjustment (*P* > .05 for all indices). As such, we can assume that the deficits in psychological adjustment over time are unlikely to be attributable to altered attention to anticipated distress responses.

## Conclusions

Our results have important implications for the news media and emergency management and public health officials. That prestorm psychological factors have a stronger association than perceived evacuation zone status or direct hurricane exposure with storm-related media consumption and subsequent adjustment suggests a need to improve hurricane-related risk communications for the public. Communicating a hazard-specific appropriate level of risk could mitigate this concern by ensuring that sensationalized reports are not creating undue levels of prestorm stress in the population, which may contribute to more negative expectations about subsequent psychological responses. Furthermore, forecasted PTS responses may be malleable in the prestorm period, presenting an important inflection point for potential intervention. Emergency management personnel could leverage public service announcements or other education efforts to inform the public about the potential risks of exposure to sensationalized media coverage. As climate scientists predict more active Atlantic hurricane seasons, it is especially important that we consider ways to mitigate the psychological risks that accompany the increasing frequency and intensity of hurricanes in coastal communities.

## References

[1] Galea S, Nandi A, Vlahov D The epidemiology of post-traumatic stress disorder after disasters. Epidemiol Rev. 2005;27:-. doi:10.1093/epirev/mxi003 15958429

[2] Norris FH, Friedman MJ, Watson PJ, Byrne CM, Diaz E, Kaniasty K 60,000 disaster victims speak, part I: an empirical review of the empirical literature, 1981-2001. Psychiatry. 2002;65(3):207-239. doi:10.1521/psyc.65.3.207.20173 12405079

[3] Galea S, Brewin CR, Gruber M, Exposure to hurricane-related stressors and mental illness after Hurricane Katrina. Arch Gen Psychiatry. 2007;64(12):1427-1434. doi:10.1001/archpsyc.64.12.1427 18056551 PMC2174368

[4] Garfin DR, Silver RC Responses to natural disasters In: Friedman HS, ed. Encyclopedia of Mental Health. Vol 4 2nd ed Waltham, MA: Academic Press; 2016:35-46. doi:10.1016/B978-0-12-397045-9.00161-0

[5] Piotrowski C, Armstrong TR Mass media preferences in disaster: a study of Hurricane Danny. Soc Behav Personal. 1998;26(4):341-346. doi:10.2224/sbp.1998.26.4.341

[6] Palen L, Hughes AL Social media in disaster communication In: Rodríguez H, Donner W, Trainor JE, eds. Handbook of Disaster Research. Cham, Switzerland: Springer International Publishing; 2018:497-518. doi:10.1007/978-3-319-63254-4_24

[7] Pfefferbaum B, Newman E, Nelson SD, Nitiéma P, Pfefferbaum RL, Rahman A Disaster media coverage and psychological outcomes: descriptive findings in the extant research. Curr Psychiatry Rep. 2014;16(9):464. doi:10.1007/s11920-014-0464-x 25064691 PMC4144190

[8] McLeish AC, Del Ben KS Symptoms of depression and posttraumatic stress disorder in an outpatient population before and after Hurricane Katrina. Depress Anxiety. 2008;25(5):416-421. doi:10.1002/da.20426 17969132

[9] Goodwin R, Palgi Y, Hamama-Raz Y, Ben-Ezra M In the eye of the storm or the bullseye of the media: social media use during Hurricane Sandy as a predictor of post-traumatic stress. J Psychiatr Res. 2013;47(8):1099-1100. doi:10.1016/j.jpsychires.2013.04.006 23673141

[10] Wilson TD, Gilbert DT Affective forecasting In: Zanna MP, ed. Advances in Experimental Social Psychology. Vol 35. San Diego, CA: Elsevier Academic Press; 2003:345-411. doi:10.1016/S0065-2601(03)01006-2.

[11] Ben-Zur H, Almog N Post-traumatic symptoms and future orientation among Israeli adolescents two years after the Second Lebanese War: the effects of war exposure, threat and coping appraisals. J Child Adolesc Trauma. 2013;6:187-200. doi:10.1080/19361521.2013.807324

[12] Holman EA, Silver RC Future-oriented thinking and adjustment in a nationwide longitudinal study following the September 11th terrorist attacks. Motiv Emot. 2005;29(4):389-410. doi:10.1007/s11031-006-9018-9

[13] Rubin DC Schema-driven construction of future autobiographical traumatic events: the future is much more troubling than the past. J Exp Psychol Gen. 2014;143(2):612-630. doi:10.1037/a0032638 23607632 PMC3778053

[14] Berntsen D, Rubin DC Pretraumatic stress reactions in soldiers deployed to Afghanistan. Clin Psychol Sci. 2015;3(5):663-674. doi:10.1177/2167702614551766 26366328 PMC4564108

[15] Gilbert DT, Pinel EC, Wilson TD, Blumberg SJ, Wheatley TP Immune neglect: a source of durability bias in affective forecasting. J Pers Soc Psychol. 1998;75(3):617-638. doi:10.1037/0022-3514.75.3.617 9781405

[16] Hoerger M, Quirk SW, Chapman BP, Duberstein PR Affective forecasting and self-rated symptoms of depression, anxiety, and hypomania: evidence for a dysphoric forecasting bias. Cogn Emot. 2012;26(6):1098-1106. doi:10.1080/02699931.2011.631985 22397734 PMC3371284

[17] Wenze SJ, Gunthert KC, German RE Biases in affective forecasting and recall in individuals with depression and anxiety symptoms. Pers Soc Psychol Bull. 2012;38(7):895-906. doi:10.1177/0146167212447242 22649114

[18] Mogg K, Millar N, Bradley BP Biases in eye movements to threatening facial expressions in generalized anxiety disorder and depressive disorder. J Abnorm Psychol. 2000;109(4):695-704. doi:10.1037/0021-843X.109.4.695 11195993

[19] Keogh E, Ellery D, Hunt C, Hannent I Selective attentional bias for pain-related stimuli amongst pain fearful individuals. Pain. 2001;91(1-2):91-100. doi:10.1016/S0304-3959(00)00422-X 11240081

[20] Lipp OV, Derakshan N Attentional bias to pictures of fear-relevant animals in a dot probe task. Emotion. 2005;5(3):365-369. doi:10.1037/1528-3542.5.3.365 16187873

[21] Heath RL, Gay CD Risk communication: involvement, uncertainty, and control’s effect on information scanning and monitoring by expert stakeholders. Manage Commun Q. 1997;10(3):342-372. doi:10.1177/0893318997010003004

[22] National Oceanic and Atmospheric Administration Extremely active 2017 Atlantic hurricane season finally ends: investments in forecasting and research yield more accurate predictions. https://www.noaa.gov/media-release/extremely-active-2017-atlantic-hurricane-season-finally-ends. Published November 30, 2017. Accessed July 9, 2018.

[23] Cangialosi JP, Latto AS, Berg R National Hurricane Center tropical cyclone report: Hurricane Irma. https://www.nhc.noaa.gov/data/tcr/AL112017_Irma.pdf. Updated June 30, 2018. Accessed July 31, 2018.

[24] Mottishaw LL The media is entertaining us with Irma, not informing us. See the difference. The Knife. https://www.theknifemedia.com/world-news/if-youre-looking-to-the-media-to-follow-irma-good-luck/. Published September 11, 2017. Accessed July 31, 2018.

[25] Deb S As Irma’s winds rise, so does a debate over TV storm reporting. *New York Times* September 10, 2017:A16.

[26] Holman EA, Garfin DR, Silver RC Media’s role in broadcasting acute stress following the Boston Marathon bombings. Proc Natl Acad Sci U S A. 2014;111(1):93-98. doi:10.1073/pnas.1316265110 24324161 PMC3890785

[27] Prins A, Bovin MJ, Smolenski DJ, The Primary Care PTSD Screen for *DSM-5* (PC-PTSD-5): development and evaluation within a veteran primary care sample. J Gen Intern Med. 2016;31(10):1206-1211. doi:10.1007/s11606-016-3703-5 27170304 PMC5023594

[28] Derogatis LR BSI 18, Brief Symptom Inventory 18: Administration, Scoring and Procedures. Minneapolis, MN: NCS Assessments; 2001.

[29] Derogatis LR Brief Symptom Inventory: Administration and Procedures Manual–I. Baltimore, MD: Clinical Psychometric Research, Inc; 1982.

[30] Ware JE Jr, Sherbourne CD The MOS 36-Item Short-Form Health Survey (SF-36), I: conceptual framework and item selection. Med Care. 1992;30(6):473-483. doi:10.1097/00005650-199206000-00002 1593914

[31] Silver RC, Holman EA, McIntosh DN, Poulin M, Gil-Rivas V Nationwide longitudinal study of psychological responses to September 11. JAMA. 2002;288(10):1235-1244. doi:10.1001/jama.288.10.1235 12215130

[32] Holman EA, Silver RC, Poulin M, Andersen J, Gil-Rivas V, McIntosh DN Terrorism, acute stress, and cardiovascular health: a 3-year national study following the September 11th attacks. Arch Gen Psychiatry. 2008;65(1):73-80. doi:10.1001/archgenpsychiatry.2007.6 18180431

[33] Bollen KA, Tueller SJ, Oberski D Issues in the structural equation modeling of complex survey data. In: Proceedings of the 59th World Statistics Congress 2013. Hong Kong: International Statistical Institute; 2013.

[34] Holman EA, Silver RC Health status and health care utilization following collective trauma: a 3-year national study of the 9/11 terrorist attacks in the United States. Soc Sci Med. 2011;73(4):483-490. doi:10.1016/j.socscimed.2011.06.018 21839560

[35] Loftus EF, Miller DG, Burns HJ Semantic integration of verbal information into a visual memory. J Exp Psychol Hum Learn. 1978;4(1):19-31. doi:10.1037/0278-7393.4.1.19 621467

[36] Schmolck H, Buffalo EA, Squire LR Memory distortions develop over time: recollections of the O.J. Simpson trial verdict after 15 and 32 months. Psychol Sci. 2000;11(1):39-45. doi:10.1111/1467-9280.00212 11228841

[37] Jones NM, Thompson RR, Dunkel Schetter C, Silver RC Distress and rumor exposure on social media during a campus lockdown. Proc Natl Acad Sci U S A. 2017;114(44):11663-11668. doi:10.1073/pnas.1708518114 29042513 PMC5676907

