# Reversed Arrows in Figure 2

**DOI:** 10.1001/jamanetworkopen.2019.0011

**Published:** 2019-01-18

In the article titled “Media Coverage, Forecasted Posttraumatic Stress Symptoms, and Psychological Responses Before and After an Approaching Hurricane,”^1^ 2 arrows in Figure 2 were reversed on initial publication. The arrow between “Irma-related media exposure” and “Wave 2 adjustment,” as well as the arrow between “Degree of hurricane exposure” and “Wave 2 adjustment,” were previously pointing away from “Wave 2 adjustment” and are now pointing toward. This article has been corrected online.
